# The Role of Non-Coding RNAs in Kidney Diseases

**DOI:** 10.3390/ijms23126624

**Published:** 2022-06-14

**Authors:** Laurent Metzinger, Juan Antonio Moreno, Valérie Metzinger-Le Meuth

**Affiliations:** 1 HEMATIM UR 4666, C.U.R.S, Université de Picardie Jules Verne, CEDEX 1, 80025 Amiens, France; valerie.metzinger@univ-paris13.fr; 2 Maimonides Biomedical Research Institute of Cordoba (IMIBIC), Hospital Universitario Reina Sofía, 14004 Cordoba, Spain; juan.moreno@imibic.org; 3 Biomedical Research Networking Center on Cardiovascular Diseases (CIBERCV), 28029 Madrid, Spain; 4 Department of Cell Biology, Physiology and Immunology, University of Cordoba, 14071 Cordoba, Spain; 5 INSERM UMRS 1148: Laboratory for Vascular Translational Science (LVTS), UFR SMBH, Université Sorbonne Paris Nord, CEDEX, 93017 Bobigny, France

Renal diseases include different pathologies, such as acute kidney injury (AKI), chronic kidney disease (CKD), end-stage renal disease (ESRD), diabetic nephropathy (DN), kidney cancer, polycystic kidney disease, etc. In recent years, the incidence and prevalence of renal diseases is increasing worldwide. These pathologies are associated with high morbidity and mortality, mainly cardiovascular [1]. There is a need for new biomarkers in kidney diseases, especially when symptoms are not detectable. They may provide innovative diagnostic and prognostic information. This Special Issue was developed to share more recent progresses in the non-coding RNA field related to renal diseases. Non-coding RNAs are transcripts that are not translated into protein, and are divided in two big classes: long non-coding RNAs and short non-coding RNAs. These include the much-studied microRNAs (miRNAs) and small interfering RNAs, but also the lesser known Piwi-interacting RNAs, small nucleolar RNAs, and other short RNAs. In addition to their potential roles as biomarkers, non-coding RNAs are groundbreaking new therapeutic approaches in the nephrology field as they can modulate different processes involved in the onset and progression of kidney diseases. 

Several strategies, such as microvesicles, high-density lipoproteins, and exosomes, have been shown to deliver functional miRNAs alongside other compounds (mRNAs, lipids, proteins, etc.). These naturally occurring nanomaterials can be used to deliver functional lncRNAs, miRNAs, or antisense sequences to the target cells. Artificial nanotechnologic particles (e.g., gold nanoparticles) can also carry therapeutic RNA molecules in diseased tissues, including the kidney [2].

DN is a serious complication of diabetes and is a common cause of ESRD. Chronic exposure to high glucose levels triggers renal oxidative stress and cell senescence. Ubc13-catalyzed K63 ubiquitination is a major control point for immune signaling that also promotes epithelial–mesenchymal transition and progression towards tubular fibrosis in DN. Pontrelli et al. [3] studied ubiquitination (more precisely its inhibition) in both cellular and murine DN models. They showed that the specific E2 complex inhibitor compound NSC697923, alone or in combination with the renin–angiotensin aldosterone system inhibitor ramipril, decreased hyperglycemia-induced epithelial–mesenchymal transition by significantly reducing the accumulation of K63-linked polyubiquitin chains. Interestingly, they also demonstrated that the tubulointerstitial accumulation of K63-ubiquitinated proteins was correlated with a decrease in the expression of miR-27b-3p in urine, whereas treated mice recovered normal urinary miR-27b-3p and levels. The authors concluded that the selective inhibition of K63 ubiquitination, in combination with other inhibitors, might be an innovative approach to inhibit the progression of fibrosis and proteinuria in DN, and suggested that miR-27b-3p may be a suitable biomarker of treatment efficiency in this pathological context.

The research article of Diaz et al. [4] analyzed the role of the miR-7641 during the course of peritoneal hyalinizing vasculopathy (PHV), a phenomenon that occurs during long-term peritoneal dialysis (PD). In a cross-sectional study, the authors did a miRNA-specific RNA-Seq analysis in a cohort of 100 non-selected peritoneal biopsies of PD patients. Peritoneal biopsies from PHV patients exhibited a loss of endothelial markers and increased collagen expression, indicating an activation of the TGF-β1/Smad3 signaling pathway that was associated with increased levels of miR-7641. The authors suggested that the endothelial–mesenchymal transition process takes place in the PHV process. This confirms previous results showing that miR-7641 was downregulated during differentiation from human embryonic stem cells to endothelial cells [5].

Peters et al. [6] highlighted the important roles of four miRNAs, miR-103a-3p, mir-192-5p, miR-29 family, and miR-21-5p in chronic kidney disease (CKD), with a focus on cardiovascular disorders linked to CKD. They point out that these miRNAs have a clear potential for clinical application in the next future as miR-21-5p is heavily involved in diabetic nephropathy and anti-miR-103a-3p has been shown to be useful in hypertensive nephropathy. They conclude that human clinical trials based on specific miRNA targeting are clearly needed to evaluate their potential therapeutic value.

Fourdinier et al. [7] analyzed the circulating levels of miR-126 in a large cohort of CKD patients [8] at all stages of the disease. They found for the first time that serum miR-126 concentration correlated significantly with the levels of the endothelial dysfunction biomarker Syndecan-1, as well as different uremic toxins, such as free indoxyl sulfate and total p-cresyl glucuronide. In their study, the authors suggested the potential role of miR-126 in endothelial dysfunction, although no molecular mechanisms have yet been found. Further studies are necessary to understand this interesting association.

Moreno et al. [9], Zhou and Li [10], and Srivastava [11] provided reviews on the roles of miRNAs, but also of their longer counterparts lncRNAs in the nephrological field. Since the discovery of most lncRNAs is more recent than the one of miRNAs, they have of course been studied less. Moreno et al. [9] focused on the molecular mechanisms involved in other renal disorders, such as AKI, CKD, and DN, as well as kidney disorders, due to toxic agents. In their respective reviews, the authors exposed lncRNA roles as innovative prognostic biomarkers for all kidney diseases and discussed new therapeutic opportunities to diminish renal injury by targeting lncRNA with antisense oligonucleotides. Zhou and Li [10] particularly focused on the roles of lncRNAs in genetically inherited kidney disorders, such as autosomal dominant polycystic kidney disease (ADPKD), which is caused by mutations of in the *PKD1* or *PKD2* genes. They also discussed the usefulness of miRNAs as innovative biomarkers in other genetic diseases, such as Alport syndrome, congenital abnormalities of the kidney and urinary tract (CAKUT), von Hippel–Lindau (VHL) disease, and Fabry disease. Srivastava [11] reported the role of miRNAs and lncRNAs interactions on DN progression and indicated that the complex crosstalk between these molecules modulates the expression of key genes involved in pathological mechanisms associated to DN, such as fibrogenesis, ER stress, inflammation, oxidative stress, and metabolic dysfunction. Therefore, lncRNAs may be potential therapeutic targets against diabetic complications including DN.
